# AAPM medical physics practice guideline 7.a.: Supervision of medical physicist assistants

**DOI:** 10.1002/acm2.12774

**Published:** 2019-12-04

**Authors:** J. Anthony Seibert, Anthony P. Blatnica, Jessica B. Clements, Per H. Halvorsen, Michael G. Herman, Jennifer L. Johnson, Beth A. Schueler, Melissa C. Martin, Jatinder R. Palta, Douglas E. Pfeiffer, Robert J. Pizzutiello, Joann I. Prisciandaro, George W. Sherouse

**Affiliations:** ^1^ UC Davis Health Sacramento CA USA; ^2^ Tufts Medical Center Boston MA USA; ^3^ Southern California Permanente Medical Group Los Angeles CA USA; ^4^ Lahey Health Burlington MA USA; ^5^ Mayo Clinic Rochester MN USA; ^6^ Kelsey-Seybold Clinic Houston TX USA; ^7^ Mayo Clinic Rochester MN USA; ^8^ Therapy Physics Inc. Signal Hill CA USA; ^9^ Virginia Commonwealth University Richmond VA USA; ^10^ Boulder Community Health Boulder CO USA; ^11^ Instrument for Change, Inc Victor NY USA; ^12^ Unversity of Michigan Ann Arbor MI USA; ^13^ Sherouse Medical Physics Durham NC USA

**Keywords:** medical physicist assistant, medical physics practice guidelines, MPA supervision, QMP‐MPA ratios, qualified medical physicist

## Abstract

The American Association of Physicists in Medicine (AAPM) is a nonprofit professional society whose primary purposes are to advance the science, education and professional practice of medical physics. The AAPM has more than 8,000 members and is the principal organization of medical physicists in the United States.

The AAPM will periodically define new practice guidelines for medical physics practice to help advance the science of medical physics and to improve the quality of service to patients throughout the United States. Existing medical physics practice guidelines will be reviewed for the purpose of revision or renewal, as appropriate, on their fifth anniversary or sooner.

Each medical physics practice guideline represents a policy statement by the AAPM, has undergone a thorough consensus process in which it has been subjected to extensive review, and requires the approval of the Professional Council. The medical physics practice guidelines recognize that the safe and effective use of diagnostic and therapeutic radiology requires specific training, skills, and techniques, as described in each document. Reproduction or modification of the published practice guidelines and technical standards by those entities not providing these services is not authorized.

The following terms are used in the AAPM practice guidelines:

- Must and Must Not: Used to indicate that adherence to the recommendation is considered necessary to conform to this practice guideline.
- Should and Should Not: Used to indicate a prudent practice to which exceptions may occasionally be made in appropriate circumstances.

Approved by AAPM’s Executive Committee May 28, 2019

## 1 Introduction

The design and management of a medical physics practice within a healthcare organization must be principally the responsibility of a Qualified Medical Physicist (QMP). Taking into account local circumstances and resources, there may be certain tasks within the program that the QMP determines can be assigned to a Medical Physicist Assistant (MPA) under the QMP's supervision as described in AAPM Professional Policy 29.^1^ Such assignment does not absolve the QMP of legal, ethical or other professional responsibility for the quality of the medical physics practice. The assigned task is in all ways the responsibility of the QMP.

This practice guideline establishes guidance for assigning medical physics‐related tasks and appropriate supervision levels for an MPA performing medical physics‐related tasks. This document does not address the levels of supervision necessary for the clinical training of medical physics students, residents, and medical physicists‐in‐training. This information can be found in MPPG 3.a. *Levels of Supervision in Clinical Medical Physics.* 2 Experience gained as an MPA achieved with on‐the‐job training is not consistent with the experience requirements and defined pathway for becoming a QMP.

It is the responsibility of all healthcare staff to be familiar with the federal and state regulations that may take precedence over the recommendations in this document. It must also be recognized that billing for professional and technical services often entails an implied or explicit requirement that the services are provided by a qualified medical physicist; failure to provide the appropriate level of QMP engagement is unethical^3^, ^4^, ^5^ and may be deemed illegal.

## 2 Definitions

- Competency –The demonstrated and documented ability to perform the assigned task or function independently.
- Medical Physicist Assistant (MPA) – An individual (e.g., radiologic technologist, medical dosimetrist, field service engineer) performing assigned tasks under the supervision and responsibility of a QMP. Expected requirements are defined by AAPM Professional Policy 29.^1^ An individual who is on an education and training pathway to become a board‐certified medical physicist may not be eligible to also contemporaneously work as an MPA if superseding restrictions are imposed by the training program or other authority. The term Medical Physicist Assistant is used in this document as a broad term, meant to comprise all employment situations in which an individual who is not a QMP extends a QMP through a formal chain of authority. While the job title in some situations and types of practices may indeed be Medical Physicist Assistant, it is possible that local specifics of the employment environment might make it appropriate to use a different title. The term MPA within this document should be read as referring to a class of extender, not to any specific job title.
- Qualified Medical Physicist (QMP) ‐ As defined by AAPM Professional Policy 1.^2^
- Signing ‐ The process whereby a QMP reviews, documents and formally assumes responsibility for the tasks performed by the MPA and the resulting work product.
- Supervision ‐ Oversight of and acceptance of responsibility for the medical physics‐related work performed by an MPA:
  - General Supervision – The medical physics activity is performed under a QMP’s overall direction and control but the QMP’s presence is not required during the performance of the medical physics activity (e.g., survey, testing or data collection). Under General Supervision, the training of the personnel who actually perform the medical physics activity and the maintenance of the necessary equipment and supplies are the continuing responsibility of the QMP.
  - Direct Supervision – A QMP must exercise the direction, control and training specified under General Supervision and be physically present in the facility and immediately available to furnish assistance and direction throughout the performance of the procedure. It does not mean that the QMP must be present in the room when the procedure is being performed.
  - Personal Supervision – A QMP must exercise the direction, control and training specified under General Supervision and be physically present in the room during the performance of the procedure.
- Supervision Plan – A written document describing tasks assigned to the MPA and the required supervision level for each task.
- Supervisor – A QMP who assumes responsibility for the medical physics‐related work of an MPA in a clinical environment.
- Task – The action or process performed by an MPA under a level of supervision defined by a QMP.
- Work Product – A deliverable or outcome that must be produced as part of the clinical work to complete a project and achieve its objectives.

## 3 Education and Training

The QMP has responsibility for determining and justifying that the education and training requirements of the MPA are commensurate with the task(s) assigned, while ensuring compliance with regulatory mandates and accreditation requirements. Education and training requirements may vary widely depending on the assigned tasks. An MPA who supports a large digital data repository may require education and training in informatics or database programming, for example, whereas an MPA who performs clinical machine quality control tasks may require education and training as a radiation therapist, nuclear medicine technologist or ultrasonography technician depending on the type of clinical machines supported. The AAPM’s Professional Policy 29^1^ defines educational requirements for MPAs. It is expected that those using this document will be familiar with and follow the provisions of all relevant AAPM guidance related to the types of MPA‐assigned tasks.

## 4 The Responsibilities of the QMP

The essential responsibility of the QMP’s clinical practice is to assure the safe and effective use of radiation (ionizing, nonionizing), radiofrequency, ultrasound or magnetic fields to achieve a diagnostic or therapeutic result as prescribed in patient care. The QMP establishes the quality program necessary to achieve this objective. The Scope of Practice of clinical medical physics is defined in AAPM Professional Policy 17.^4^ MPPG 10, Scope of Practice for Clinical Medical Physics,^6^ specifies the tasks that must be performed by a QMP; these tasks must not be assigned to an MPA. MPPG 10 also describes the level of supervision to be provided to MPAs for tasks that may be assigned.

A QMP must not assign any task to an MPA when such assignment could potentially increase the risk of harm to the patient, personnel, or the public due to the MPA’s lack of qualifications or competence. In determining if a task can be assigned, the QMP takes into account the likelihood of error, the severity of the impact of potential error, and the likelihood that an error would go undetected. In addition, all of the following criteria must be met:

- The MPA has demonstrated competence, documented by the QMP, for the specific task.
- The task can be performed independently of other tasks (i.e., not requiring intricate, complex knowledge of parallel or subsequent processes to adequately perform the assigned task).
- The task must be sufficiently routine that requisite knowledge and experience do not demand that the QMP actually performs the task, but rather a review by the QMP establishes the same level of confidence in the end result.
- For data collection tasks, the QMP has performed baseline measurements and established protocols for the specific piece of equipment on which the MPA will be collecting data.
- The MPA complies with the supervision plan determined by the QMP.

A supervision plan must be developed by the QMP in accordance with the AAPM Scope of Practice for Clinical Medical Physics.^6^ The supervision plan must outline the tasks, describe the level of supervision (e.g., general, direct, or personal), provide the rationale for each task assigned to the MPA, and be structured to ensure regular, direct contact between the supervising QMP and each MPA. It should explicitly include minimum frequency of QMP/MPA interaction and the timeline for QMP review of MPA work products. The MPA must not be assigned tasks beyond documented qualifications and competency. The supervision level must be based on the risk level of the task and the competency level of the MPA. Sample supervision plans are provided in Appendices S1–S3.

Ongoing supervision of an MPA must be formally structured in a way that explicitly maintains the personal accountability of the QMP for all work and work product, while assuring that the MPA has ample opportunity to receive guidance and advice on the performance of the work. Direct conversation on an agreed frequency is an important element of any supervision plan and is particularly crucial for work that is largely performed under general supervision. The scope of these personal interactions should extend beyond the technical details of the work to include bidirectional sharing of information and concerns regarding the environment in which the MPA extends the QMP, for instance information regarding changes in the hospital’s personnel, practices or equipment. Determining the nature and frequency of these personal interactions as appropriate to each item of assigned work, as well as enforcement of the interaction schedule, is the responsibility of the QMP. Regularly scheduled face‐to‐face meetings and more frequent direct conversations are necessary. Ongoing interactions notwithstanding, the QMP **must** review all tasks and sign all supervised work products of the MPA. Under no circumstances can the authority to sign the work product of an MPA that requires QMP review be assigned to anyone other than a duly designated QMP. The record must bear the signature of both the MPA who performed and documented the work, and the QMP who accepts responsibility for its quality.

During a supervising QMP’s absence (e.g., sick leave, extended absence in accordance with the Family Medical Leave Act, maternity/paternity leave) the supervising QMP is responsible for documenting the delegation of the required supervision of the MPA to a QMP providing coverage for the supervising QMP. In the event that the supervising QMP vacates the medical physics practice, the vacating QMP shall notify the facility administrator that the MPA must be properly supervised by a QMP in order to continue performing the assigned tasks.

## 5 The Responsibilities of the Medical Physicist Assistant

The MPA performs the tasks assigned in accordance with an approved supervision plan protocols and procedures established by the QMP. The MPA is responsible for the integrity of the data collected and work performed. The MPA may perform assigned tasks in an established medical physics practice (e.g., periodic checks after a QMP has performed acceptance testing and commissioning and has established a baseline). The MPA must seek assistance from the QMP in any situation presenting unexpected results or unusual circumstances. MPA tasks must be in compliance with federal and state regulations and accreditation requirements.

The MPA must not accept a task that is outside the approved supervision plan, or otherwise agree to a supervision plan that requires tasks outside the MPA's documented qualifications and competency. The MPA must not present as a QMP, or as capable of practicing medical physics independently. The MPA must not provide technical advice outside assigned tasks. If a supervising QMP vacates the medical physics practice, the MPA must not perform any work detailed in the supervision plan and must inform the facility administrator.

The traditional role of the QMP – MPA supervision may change over time with advances in technology and scope of work. It is the responsibility of the MPA to communicate with the supervising QMP to ensure appropriate that oversight and documentation are achieved when nontraditional (future) tasks not in the current supervision plan are proposed.

The MPA should have training and awareness of safety culture and health system standard precautions from infectious diseases and other hazards for self‐protection and protection of others, including patients. Assimilation into a clinical safety and reporting culture mindset is an ethical responsibility of the MPA that should be coordinated with the QMP and the employer.

## 6 The Responsibilities of the Facility Administrator

Facility administrators are advised to review the use of an MPA with the managing QMP in accordance with this practice guideline. The facility administrator must understand that the MPA may not work independently, nor provide work product that is not signed by a properly designated QMP.

In the event that a supervising QMP vacates the medical physics practice or is incapacitated, the facility administrator must ensure proper supervision by an appropriate alternate QMP, according to the supervision plan of either the previous or current QMP.

## 7 Staffing

Medical physics support encompasses much more than just the rote performance of specific tasks. Many clinical tasks must be performed by a QMP. Therefore, appropriate supervision of an MPA for assigned tasks is a significant commitment. For these reasons, there must be a limit to the number of MPAs supervised by a QMP.

The nature of the work, variations in practice environments, and the levels of risk are substantially different between therapeutic and diagnostic practices. The procedure‐specific work of a radiotherapy physicist is paid by the patient or an insurer on the assumption that the work will be performed by a QMP. In that context, for both legal and ethical reasons, care must be taken to assure that the use of MPAs does not deprive the radiotherapy patient of the personal attention of a QMP for which the patient ultimately is paying. Typically, the majority of the work of a radiotherapy physicist is in support of a CPT‐defined patient procedure and cannot be assigned to a non‐QMP. This motivates the recommendation of a maximum 0.25:1 Full Time Equivalent (FTE) ratio of MPAs to QMPs. Furthermore, it is inappropriate to use an MPA in a practice setting with less than 1 FTE QMP per location. The MPA must have access on a daily basis to the supervising QMP.

The current practices of diagnostic and nuclear medical physics differ from that of therapy medical physics. In these practices, some routine tasks can be assigned to MPAs, but the scientific expertise and experience of the QMP is essential to ensure the quality and safety of the practice. Thus, a nominal ratio of 1:1 for MPAs to QMPs should be observed for Diagnostic and Nuclear Medical Physics. As there exist substantial differences between different practice settings, other ratios may be used with documented justification. The justification should be based on prudent risk assessment given the nature of the assigned task, the skill set of the MPA and the QMP's ability (geography, workload) to properly supervise and to be available for support. Regardless of the ratio, the supervising QMP is held directly responsible for the quality of the physics service and should exercise frequent supervision over all MPA activities. FTE ratios greater than 1:1 should be considered cautiously and in no case should exceed 4 MPAs per 1 QMP. Other ratios may be used, provided a clear determination of need is presented, in conjunction with documented justification. The supervising QMP must have one‐on‐one contact with each MPA on a routine and frequent basis.

AAPM recommends a supervision ratio as outlined in Table 1.

**Table 1:** Medical Physicist Assistant Supervision Ratios

Practice	Maximum FTE MPA per clinical FTE QMP
Diagnostic Medical Physics	1
Nuclear Medical Physics	1
Therapeutic Medical Physics	0.25

## 8 Competency

The QMP designs and implements a formal and well‐documented structure for the MPA to demonstrate the ability to fulfill each task to be assigned. Task‐specific expectations of competency must be formally defined in writing by the QMP for each assigned task. The MPA must demonstrate the ability to *consistently, correctly and accurately* perform each task, and must therefore perform the task under the QMP’s personal supervision in order to establish competence to the satisfaction of the QMP. Documentation of competence for each assigned task must be maintained by the QMP, and competence should be reviewed at least annually through personal supervision. Individuals who have not yet formally demonstrated competency in specific tasks must be under the personal supervision of the QMP. If periodic review through audit or examination reveals an erosion of competency, then an adjustment of the supervision plan is indicated until such time as the competency can be re‐established.

## 9 Conclusions

Clinical practice environments must balance the need for competent staff with the need to provide services in a cost‐effective yet safe manner. Considering this practice guideline, QMPs are advised to assess the use of an MPA in their medical physics practice and implement appropriate changes. Therefore, the following considerations must be taken into account:

1. When an organization employs an MPA, it is the responsibility of the QMP to inform the healthcare organization of the need for a supervision plan and its requirements.
2. Facility administrators are advised to review the use of an MPA with the managing QMP.
3. A QMP must only assign tasks having low risk of harm to the patient, personnel, and the public consistent with MPPG 10, Scope of Practice for Clinical Medical Physics.^6^
4. The supervision level for the assigned task must be based on the risk level of the task and the competency level of the MPA.

## Conflict of Interest

AAPM members are required to update and reattest to a personal conflict of interest statement annually. All authors have up to date AAPM conflict of interest statements, available on their member profile, at the time of publication.

## Supporting information


**Appendix S1**. (a) SAMPLE 1 ‐ Supervision Plan for Therapy Medical Physicist Assistant. (b) SAMPLE 2‐ Supervision Plan for Therapy Medical Physicist Assistant.
**Appendix S2**. SAMPLE Supervision Plan for a Medical Physicist Assistant in Nuclear Medicine to Perform Annual Gamma Camera Evaluations.
**Appendix S3**. SAMPLE Supervision Plan for a Medical Physicist Assistant in Diagnostic Imaging to Perform Annual Radiographic System Evaluations.
