# Supplementary material for: AAPM medical physics practice guideline 7.a.: Supervision of medical physicist assistants
Source: J Appl Clin Med Phys. 2019 Dec 4;21(7):11–5. doi: 10.1002/acm2.12774 (PMC7386193; doi:10.1002/acm2.12774)
Supplement: Supplementary file 1 — Appendix S1. (a) SAMPLE 1 ‐ Supervision Plan for Therapy Medical Physicist Assistant. (b) SAMPLE 2‐ Supervision Plan for Therapy Medical Physicist Assistant. Appendix S2. SAMPLE Supervision Plan for a Medical Physicist Assistant in Nuclear Medicine to Perform Annual Gamma Camera Evaluations. Appendix S3. SAMPLE Supervision Plan for a Medical Physicist Assistant in Diagnostic Imaging to Perform Annual Radiographic System Evaluations. [file ACM2-21-11-s001.docx]

The following appendices are sample supervision plans. They are neither exclusive nor comprehensive, in recognition of the fact that such plans should be local products. Each discipline has a wide variety of supervision plans that should be considered. In keeping with this, the sample plans differ in structure and approach rather than standardizing them.

**Appendix 1A: SAMPLE 1 - Supervision Plan for Therapy Medical Physicist Assistant**

A qualified medical physicist provides supervision of the medical physicist assistant(s).

Supervision plan:

The supervisor is responsible for overseeing the training of the medical physicist assistant, assigning duties, assessing performance, and evaluating competence during transition from personal to direct or general supervision in assigned duties.

The medical physicist assistant is expected to:

1. review documentation regarding standard operating procedures of all equipment to be used,
2. learn about the safety hazards regarding any equipment that will be used such as linear accelerators, detectors (e.g., ionization chambers, diodes, arrays), survey meters and treatment planning software,
3. learn how to operate equipment such as detectors, detector arrays, and electrometers,
4. observe proper use of the equipment and learn how to troubleshoot any problems with the equipment, and
5. perform compliance training as required by the institution.

The supervisor will confirm that the medical physicist assistant understands:

1. proper use and storage of equipment,
2. how to document service activities,
3. that work product will be regularly reviewed by a qualified medical physicist, and
4. the MPA accepts responsibility for notifying the QMP when unexpected results are encountered.

**Table A1.A Sample – Detailed Therapy Medical Physicist Supervision Plan**

| **Sample – Detailed Therapy Medical Physicist Supervision Plan** | | | | | |
| --- | --- | --- | --- | --- | --- |
|  |  | Initial/Date | | |  |
|  | Knowledge factors | Not Competent | Marginally Competent | Fully Competent | Notes |
| 1 | Demonstrate an understanding of the risks and hazards presented by radiation and high voltage related to the equipment that is routinely used in the department. |  |  |  |  |
| 2 | Demonstrate an understanding of the purpose of IMRT QA, including how and why a dose comparison is performed pre-treatment between the planning system and phantom measurements. |  |  |  |  |
| 3 | Discuss and demonstrate an understanding of the equipment used to perform IMRT QA, including phantoms and the dosimetric devices (e.g., multi-diode array, film, EPID, ion chamber, electrometer etc.). |  |  |  |  |
| 4 | Demonstrate an understanding of patient-specific dosimetric measurements required for IMRT QA. |  |  |  |  |
| 5 | Demonstrate an understanding of the dose calculations routinely used in the clinic for IMRT treatment planning. |  |  |  |  |
| 6 | Discuss and review the mechanical and safety-related periodic QA for radiation therapy equipment (hardware and software). |  |  |  |  |
| 7 | Demonstrate an understanding of how to document QA and what is appropriate for an MPA to perform and what requires direct supervision of a QMP. |  |  |  |  |
| 8 | Demonstrate an understanding of what is involved in data analysis for clinical and protocol studies. |  |  |  |  |
|  |  |  |  |  |  |
|  |  | Initial/Date | | |  |
|  | Practical factors | Direct Supervision | Personal Supervision | General Supervision | Notes |
| 1 | Complete radiation and high voltage safety training. | --- | --- | --- | Date of completion: |
| 2 | Observe X phantom and equipment setup for film-based IMRT QA. |  |  | --- |  |
| 3 | Observe X phantom and equipment setup for ion chamber/point-based IMRT QA. |  |  |  |  |
| 4 | Observe X phantom and equipment setup for multi-diode array-based IMRT QA. |  |  |  |  |
| 5 | Observe X phantom and equipment setup for EPID-based IMRT QA. |  |  |  |  |
| 6 | Observe X number of patient-specific dosimetric measurements required for IMRT QA. |  |  |  |  |
| 7 | Observe X number of monthly mechanical and safety related QA for radiation therapy related equipment and software. |  |  |  |  |
| 8 | Perform quality assurance checks for patient brachytherapy procedures. |  |  |  |  |

Appendix 1B. **SAMPLE 2- Supervision Plan for Therapy Medical Physicist Assistant**

**Medical Physicist Assistant Supervision Plan**

**Policy**

The design and management of the Radiation Oncology department’s medical physics practice is the responsibility of Chief Physicist. Certain tasks may be delegated to a Medical Physicist Assistant (MPA) working under the supervision of a Qualified Medical Physicist (QMP). The delegated task remains the professional responsibility of the QMP.

The supervisor is responsible for overseeing the training of the MPA, assigning duties, assessing the MPA’s performance, and evaluating competence as the MPA transitions from personal to direct and/or general supervision in the assigned duties.

The medical physicist assistant is expected to:

- Review documentation regarding standard operating procedures of all equipment to be used
- Learn about the safety hazards regarding any equipment that will be used such as linear accelerators, CT scanners, detectors (e.g., ionization chambers, diodes, arrays), and electrometers
- Learn how to operate aforementioned equipment
- Observe proper use of the equipment and learn how to troubleshoot any problems with the equipment
- Perform compliance training as required by the institution.

The supervisor will confirm that the medical physicist assistant understands:

- Proper use and storage of equipment
- How to document work activities
- That work product will be regularly reviewed by a QMP.

**Documentation**

The Chief Physicist will review and attest to the competency of the MPA in performing the assigned tasks. This will consist of initial training and annual performance evaluations.

**MPA Initial Training Checklist**

**Medical Physicist Assistant:** Click here to enter text.

| **Task Description** | **Further training needed** | **Competent** | **Supervision level: Direct / General** | **Date** | **QMP Assessor** |
| --- | --- | --- | --- | --- | --- |
| Demonstrate understanding of risks/hazards with radiation and electromechanical components |  |  |  |  |  |
| Demonstrate competence in manufacturing electron cutouts and logistical coordination |  |  |  |  |  |
| Demonstrate familiarity with layout/conventions in each procedure room (linacs, CT-sim) in Burlington |  |  |  |  |  |
| Demonstrate familiarity with layout/conventions in each procedure room (linacs, CT-sim) in Winchester |  |  |  |  |  |
| Demonstrate competence in Linac mechanical QC tests (MQA) |  |  |  |  |  |
| Demonstrate competence in patient specific IMRT QA using MapCheck2 |  |  |  |  |  |
| Demonstrate competence in patient specific IMRT QA using Portal Dosimetry |  |  |  |  |  |
| Demonstrate competence in Novalis 6SRS flatness/symmetry MQA test |  |  |  |  |  |
| Demonstrate familiarity with file management practices (Therapy Physics) |  |  |  |  |  |
| Demonstrate competence with instrument intercomparisons and calibration status tracking |  |  |  |  |  |
| Demonstrate competence with HDR SGT MQA |  |  |  |  |  |
| Demonstrate competence with advanced Excel functions and templates |  |  |  |  |  |
| Demonstrate competence with CT-simulator MQA tests and CT-density phantom scanning |  |  |  |  |  |

**I attest that I have received the training necessary to competently perform my duties as a Medical Physicist Assistant and that I understand and will comply with the supervision plan described above.**

____________________________________ _________________

MPA signature Date

**Chief Physicist review and approval:**

____________________________________ _________________

Chief Physicist signature Date

| Contact: | **Senior Director of Radiation Oncology** |
| --- | --- |
| Origination date: | **March 2019** |
| Reviewer(s): | **Radiation Oncology Physicists** |
| Approver(s): | **Chief Physicist, Radiation Oncology** |

**Appendix 2 – SAMPLE Supervision Plan for a Medical Physicist Assistant in Nuclear Medicine to Perform Annual Gamma Camera Evaluations**

1. Introduction

The design and management of a medical physics practice in nuclear medicine within a healthcare organization must be principally the responsibility of a Qualified Medical Physicist (QMP). Taking into account local circumstances and resources, there may be certain tasks within the program that the QMP determines can be assigned to a Medical Physicist Assistant (MPA) under the QMP's supervision. Such assignment does not absolve the QMP of legal, ethical or other professional responsibility for the quality of the medical physics practice. The assigned task is always the responsibility of the QMP.

1. Supervision of a Medical Physicist Assistant

An MPA may perform assigned tasks in an established medical physics practice. MPA tasks must be in compliance with federal and state regulations and accreditation standards. Ultimately, the supervising QMP must approve and take responsibility for the tasks assigned to or performed by the MPA.

A supervision plan must be developed by the QMP. The supervision plan must outline the tasks, describe the level of supervision (e.g., general, direct or personal), and provide the rationale for each task assigned to the MPA. The assigned tasks must have a low risk of harm to the patient, personnel or public. The supervision level must be based on the risk level of the task and the competency level of the MPA.

1. Competency

The MPA must demonstrate the ability to consistently, correctly, and accurately perform each task and must therefore perform the task under the QMP’s personal supervision in order to establish competence to the satisfaction of the QMP. Competency in a task is determined when the MPA has performed the task independently with full competence at least ten times under the personal supervision of a QMP. Documentation of competence for each assigned task must be maintained by the QMP, and competence should be reviewed at least annually through personal supervision. Individuals who have not yet formally demonstrated competency in specific tasks must be under the personal supervision of the QMP. If periodic review through audit or examination reveals an erosion of competency, then an adjustment of the supervision plan is indicated until such time as the competency can be reestablished.

Nuclear medicine equipment testing and quality control competence includes:

1. performing annual compliance procedures of a nuclear medicine gamma camera, including a single-photon emission computed tomography (SPECT) system;
2. performing quality control tests and calibrations of nuclear medicine systems, dose calibrators, and counting systems;
3. monitoring the quality control program appropriate for nuclear medicine systems, dose calibrators, and counting systems, and demonstrating an understanding of the daily, monthly, quarterly, and annual tests required for each equipment type;

Documentation of task competency for annual gamma camera testing shall be maintained using the competency form appended to this document as Table 2A.

1. Rationale

Nuclear medicine gamma camera testing is a long process that can easily be interfered with by ambient radiation levels. This requires that much of the testing must occur when these ambient levels are minimized. This may require working around a busy department’s schedule. Use of an MPA provides the flexibility in scheduling that helps to minimize patient scheduling conflicts. Many of the gamma camera tasks assigned to MPAs are similar to the tasks completed by Nuclear Medicine Technologists as part of their ongoing QC program. A QMP can assign the tasks involved in gamma camera testing that have a low risk of harm to the patient, personnel, or public. This allows the QMP to focus on more complex tasks such as image quality, artifact evaluation, protocol optimization, administered activity management and patient dosimetry.

1. Independent Task Performance by the Nuclear Medicine Medical Physicist Assistant

Once the Nuclear Medicine Medical Physicist Assistant has demonstrated competency in an assigned task, these tasks can be performed independently under the general supervision of the supervising QMP. The results of all medical physics tasks performed independently by the MPA shall be documented in a report and reviewed by a Qualified Medical Physicist before it is provided to the health care facility.

1. Periodic Review of Competency

At least annually, and whenever it is determined that the medical physicist assistant may not be performing a given task satisfactorily, the competence of the MPA shall be reevaluated. This may be done under direct or personal supervision. The results of this evaluation shall be documented by the supervising QMP.

1. Supervision Level

The minimal level of supervision of a Nuclear Medicine MPA is indicated in the table below:

**Table A2.A**

| **Competency Level** | **Under Personal Supervision of QMP** | **Under Direct Supervision of QMP** | **Under General Supervision of QMP** |
| --- | --- | --- | --- |
| Initial Training | X |  |  |
| Deemed Competent |  |  | X |
| Annual Evaluation |  | X |  |
| Reevaluation of task competence when suboptimal performance of a task is suspected | X |  |  |

**Table A2.B**

| **Nuclear Medicine Medical Physicist Assistant**  **QMP Supervisor Evaluation of Competency** | | | | | |
| --- | --- | --- | --- | --- | --- |
| **MPA’s Name** |  | | | | |
| **Modality name:** | **Nuclear Medicine Gamma Camera Annual Testing** | | | | |
| **Inclusive dates of rotation:** |  | | | | |
| **Supervising QMP’s Name:** |  | | | | |
| **Evaluation criteria:** | **Not Competent** | **Marginally competent** | **Fully competent** | **Date** | **Explanatory notes** |
| **Task Skills** | **MPA must perform each task independently with full competence** | | | | |
| 1. Performs annual testing (including SPECT) of a nuclear medicine camera |  |  |  |  |  |
| 1. Performs Physical and Mechanical Inspection of Imaging Systems |  |  |  |  |  |
| 1. Performs intrinsic uniformity on each detector using clinically applicable radionuclides |  |  |  |  |  |
| 1. Performs extrinsic uniformity on each detector with each clinically applicable collimator |  |  |  |  |  |
| 1. Performs intrinsic resolution for each detector |  |  |  |  |  |
| 1. Performs spatial linearity. |  |  |  |  |  |
| 1. Performs extrinsic resolution for each detector using clinically applicable collimators |  |  |  |  |  |
| 1. Perform extrinsic sensitivity and compare to manufacturer specifications or established baselines. Evaluate relative sensitivity of both detectors/collimators |  |  |  |  |  |
| 1. Perform energy resolution and compare to manufacturer’s specifications or applicable standard |  |  |  |  |  |
| 1. Evaluate count rate parameters for each detector: |  |  |  |  |  |
| 1. Determine Maximum count rate |  |  |  |  |  |
| 1. Determine Background count rate |  |  |  |  |  |
| 1. Determine count rate at 20% data loss |  |  |  |  |  |
| 1. Determine dead time |  |  |  |  |  |
| j. Performs Center of Rotation (COR) or Axis of Rotation (AOR) on SPECT systems |  |  |  |  |  |
| k. Performs SPECT phantom imaging (Must acquire images using proper acquisition parameters, reconstruct acquired images using appropriate parameters and apply appropriate analysis of reconstructed images: |  |  |  |  |  |
| (1.) SPECT resolution |  |  |  |  |  |
| (2.) SPECT contrast |  |  |  |  |  |
| (3.) SPECT Uniformity |  |  |  |  |  |
| (4.) SPECT artifact evaluation |  |  |  |  |  |
| l. Evaluate Soft Copy Displays for applicable Acquisition Workstations (AWS) and Image Processing Workstations (IPWS): |  |  |  |  |  |
| (1.) Test Pattern Evaluation for resolution and spatial accuracy |  |  |  |  |  |
| (2.) Determine maximum luminance (L_max_) and compare to manufacturer’s specifications or other established standard |  |  |  |  |  |
| (3.) Determine minimum luminance (L_min_) and compare to manufacturer’s specifications or other established standard |  |  |  |  |  |
| (4.) Determine L_max_/L_min_ ratio and compare to manufacturer’s specifications or other established standard |  |  |  |  |  |
| (5.) Evaluate luminance uniformity and compare to established standard |  |  |  |  |  |
| 2. Perform and evaluate applicable Technologist QC tasks required by manufacturer or applicable accreditation body |  |  |  |  |  |

**Appendix 3. SAMPLE Supervision Plan for a Medical Physicist Assistant in Diagnostic Imaging to Perform Annual Radiographic System Evaluations**

A qualified medical physicist (QMP) provides supervision of the medical physicist assistant(s).

**Supervision plan:**

The QMP supervisor is responsible for overseeing the training of the medical physicist assistant, assigning duties, assessing performance, and evaluating competence during the transition from personal to direct or general supervision in assigned duties.

The medical physicist assistant is expected to:

1. review documentation regarding standard operating procedures of all equipment to be used,
2. learn about the safety hazards regarding any equipment that will be used,
3. learn how to operate testing equipment such as detectors and photometers,
4. observe proper use of the equipment and learn how to troubleshoot any problems with the equipment, and
5. perform compliance training as required by the institution.

The QMP supervisor will confirm that the medical physicist assistant must be able to:

1. perform quality control of diagnostic x-ray equipment in

accordance with state regulation, accrediting organizations, the equipment manufacturer’s standards, or as instructed by a Qualified Medical Physicist,

1. safely operate X-ray equipment,
2. operate radiation detection instrumentation and phantoms,
3. review, provide follow-up, and assist with problems detected on the routine QC test results,
4. respond to and trouble-shoot equipment performance issues,
5. identify equipment malfunctions, system hardware and software failures,
6. identify operator errors and alert the supervising QMP,
7. maintain records of routine periodic QC test results and prepares ~~monthly~~ reports.
8. maintain all instrumentation used for quality control and arrange for repairs and calibration as well as schedule and arrange for shipment,
9. coordinate equipment service events with service contractors and facility engineering team.  Follow up on service events to ensure/verify safety and proper imaging performance. Perform equipment quality assurance tests after service is completed as required,
10. assist with acceptance testing and commissioning of new equipment, and
11. maintain over the long term an appropriate level of effort and communication with Medical Physicists managing clinical physics projects to assure progress is continuous and routine quality control activities are not compromised.

**Table A3.A Sample –Diagnostic Medical Physicist Assistant Supervision Plan**

**Name: ID#: Date:**

|  | **Self Evaluation** | | **Competency Assessment** | | | | | | **Orientation / Review Comments** | **Recommendations Improvement/Action Plan and Implementation Date** |
| --- | --- | --- | --- | --- | --- | --- | --- | --- | --- | --- |
| **Skill** | **Yes** | **No** | **Date** | **Level 1** | **Level 2** | **Level 3** | **N/A** | **Initials** |  |  |
| **Modality Technical Skills** |  | | | | | | | | | |
| **General Radiography Technical Skills** |  | | | | | | | | | |
| **Understands Start Up/Shutdown procedures for:** |  | | | | | | | | | |
| CR Readers |  |  |  |  |  |  |  |  |  |  |
| Portables |  |  |  |  |  |  |  |  |  |  |
| All DR rooms located at facility |  |  |  |  |  |  |  |  |  |  |
| **Weekly Quality Assurance (QA)** |  | | | | | | | | | |
| Ability to properly perform Vendor QA tests |  |  |  |  |  |  |  |  |  |  |
| Understands the essential components of the Vendor QA tests |  |  |  |  |  |  |  |  |  |  |
| Knows the follow-up flow chart for QA failures |  |  |  |  |  |  |  |  |  |  |
| Able to effectively communicate system issues to the service department when creating a service ticket. |  |  |  |  |  |  |  |  |  |  |
| **Post Service Testing and Annual Assistance** |  | | | | | | | | | |
| Ability to use Electrometers and Acquire Data |  |  |  |  |  |  |  |  |  |  |
| Properly performs illuminance verification (Patient Positioning Lamp) test |  |  |  |  |  |  |  |  |  |  |
| Ability to Install SMPTE Pattern on applicable Platforms |  |  |  |  |  |  |  |  |  |  |
| Properly performs monitor luminance verification (SMPTE) |  |  |  |  |  |  |  |  |  |  |
| Properly performs X-Ray to Light Field Congruency test |  |  |  |  |  |  |  |  |  |  |
| Properly performs X-Ray to image receptor alignment test |  |  |  |  |  |  |  |  |  |  |
| Properly performs Collimator Dial Accuracy test |  |  |  |  |  |  |  |  |  |  |
| Properly performs Positive Beam Limitation test |  |  |  |  |  |  |  |  |  |  |
| Properly performs Sensitivity Verification for applicable CR Readers |  |  |  |  |  |  |  |  |  |  |
| Properly performs Uniformity and artifact test for applicable CR Readers and plates |  |  |  |  |  |  |  |  |  |  |
| Half-value layer measurements |  |  |  |  |  |  |  |  |  |  |
| kV accuracy |  |  |  |  |  |  |  |  |  |  |
| Exposure timer accuracy |  |  |  |  |  |  |  |  |  |  |
| Linearity and reproducibility |  |  |  |  |  |  |  |  |  |  |
| Output mR/mAs measurement |  |  |  |  |  |  |  |  |  |  |
| AEC performance |  |  |  |  |  |  |  |  |  |  |
| Artifact evaluation |  |  |  |  |  |  |  |  |  |  |

- **Level 1 – Acceptable**
- **Level II – marginal, needs improvement**
- **Level III – unacceptable, must show improvements within 6 months**

COMMENTS

Employee Signature: ____________________________________ Date: __________________________

Supervisor Signature: ____________________________________ Date: _________________________
